# The Intranasal Application of Zanamivir and Carrageenan Is Synergistically Active against Influenza A Virus in the Murine Model

**DOI:** 10.1371/journal.pone.0128794

**Published:** 2015-06-08

**Authors:** Martina Morokutti-Kurz, Marielle König-Schuster, Christiane Koller, Christine Graf, Philipp Graf, Norman Kirchoff, Benjamin Reutterer, Jan-Marcus Seifert, Hermann Unger, Andreas Grassauer, Eva Prieschl-Grassauer, Sabine Nakowitsch

**Affiliations:** 1 Marinomed Biotechnologie GmbH, Vienna, Austria; 2 Laboratory of Tropical Veterinary Medicine, Veterinary University Vienna, Vienna, Austria; Icahn School of Medicine at Mount Sinai, UNITED STATES

## Abstract

**Background:**

Carrageenan is a clinically proven and marketed compound for the treatment of viral upper respiratory tract infections. As infections caused by influenza virus are often accompanied by infections with other respiratory viruses the combination of a specific anti-influenza compound with the broadly active antiviral polymer has huge potential for the treatment of respiratory infections. Thus, the combination of the specific anti-influenza drug Zanamivir together with carrageenan in a formulation suitable for intranasal application was evaluated *in-vitro* and *in-vivo*.

**Principal Findings:**

We show *in-vitro* that carrageenan and Zanamivir act synergistically against several influenza A virus strains (H1N1(09)pdm, H3N2, H5N1, H7N7). Moreover, we demonstrate in a lethal influenza model with a low pathogenic H7N7 virus (HA closely related to the avian influenza A(H7N9) virus) and a H1N1(09)pdm influenza virus in C57BL/6 mice that the combined use of both compounds significantly increases survival of infected animals in comparison with both mono-therapies or placebo. Remarkably, this benefit is maintained even when the treatment starts up to 72 hours post infection.

**Conclusion:**

A nasal spray containing carrageenan and Zanamivir should therefore be tested for prevention and treatment of uncomplicated influenza in clinical trials.

## Introduction

The periodic appearance of new influenza variants poses a worldwide pandemic threat. Since the emergence of the new A(H7N9) virus, more than 400 human cases were reported to the WHO with a mortality rate of more than 35%. Most patients with A(H7N9) infections had contact with poultry or visited live animal markets. However, some sporadic cases seemed to be a result of human to human transmissions [1,2]. In contrast to pandemic viruses which fulminantly enter the human population and cause high mortality rates, seasonal influenza viruses generally cause uncomplicated and transient infections in humans, with virus replication localized to the upper respiratory tract [3,4]. However, in its fully developed form influenza is an acute respiratory disease resulting in hospitalizations and deaths mainly among high-risk groups. Worldwide, annual epidemics result in about three to five million cases of severe illness, and about 250,000 to 500,000 deaths [5]. For this reason WHO [6] and CDC [7] recommend antiviral treatment for any patient with suspected influenza who is at risk for influenza complications without previous laboratory confirmation.

It is known that influenza virus infections are often accompanied by other viral pathogens [8]. Depending on the detection method (qRT-PCR or immunofluorescence) different ratios of co-infections have been found. Analysis by qRT-PCR revealed that 54.5–83.3% of influenza A or B positive patients were found to have at least one concomitant respiratory viral infection [9–12]. The detection frequency with immunofluorescence was found to be even higher (90–100%) [13,14]. Potential concomitant viral pathogens of influenza virus infections include human rhinovirus (hRV), respiratory syncytial virus, adenovirus, human coronavirus, human metapneumovirus and parainfluenza virus [14,15].

As a result of the multiple infections, a specific anti-influenza mono-therapy treats the influenza virus infection only, but not the infection with the concomitant viral pathogen. Hence, the therapy often fails to sufficiently resolve symptoms. This is also reflected by the fact that neuraminidase inhibitors (NI) are highly efficacious in animal models investigating influenza mono-infections [16,17] but show lower efficacy against influenza symptoms in clinical trials in adults with natural infections [18]. Therefore, there is a high medical need for a broadly acting antiviral therapy in combination with a specific anti-influenza therapy for treatment of patients suffering from upper respiratory tract symptoms. Ideally, the substances present in the combination complement each other by different modes of action, leading to a treatment that provides full protection against a broad range of different respiratory viruses as well as different influenza strains with a low probability to induce escape mutations.

One approach for a broad antiviral therapy is the creation of a protective physical barrier in the nasal cavity using carrageenan. Carrageenan is a high molecular weight sulfated polymer derived from red seaweed (*Rhodophyceae*) that has been extensively used in food, cosmetic and pharmaceutical industry and is generally recognized as safe by the FDA (GRAS) (reviewed in [19]). Three main forms of carrageenans are commercially used: kappa, iota and lambda. They differ from each other in the degree of sulfation, solubility and gelling properties [20]. The antiviral mechanism of carrageenan is based on the interference with viral attachment; as a consequence, viral entry is inhibited [21,22]. Its antiviral activity is dependent on the type of polymer as well as the virus and the host cells [23–32] and has been reviewed in [33–35]. We published that iota-carrageenan is a potent inhibitor of hRV [36] and influenza A [37] replication and demonstrated the antiviral efficacy of iota-carrageenan against common cold viruses by intranasal application in several randomized, double-blind, parallel group, placebo-controlled clinical trials [38–40]. The pooled analysis of two studies conducted in 153 children and 203 adults revealed that patients infected with any respiratory virus, who were intranasally treated with iota-carrageenan showed a 1.9 day faster recovery from common cold symptoms than placebo treated patients in the intention-to-treat population [41,42]. The anti-influenza activity was shown by subgroup analysis of 49 influenza infected patients who benefited from a 3.3 days faster recovery from symptoms. The use of carrageenan nasal spray was associated with a significant reduction of the influenza viral load in nasal fluids and a significant increase in the number of virus free patients within the treatment period of 7 days. In good accordance with the literature [9–14] we observed that the majority of influenza virus infected patients suffered from a concomitant respiratory viral infection (66%) as determined by real-time PCR. Carrageenan containing nasal sprays are already marketed for the treatment of respiratory viral infections under different brand names in 18 countries.

At present the only available effective drugs for treatment and post exposure prevention of influenza are the NI (Oseltamivir and Zanamivir worldwide; Peramivir in Japan and South Korea). Since the large-scale use of M2 blockers for prophylaxis and treatment in humans [43] and farming [44], the currently circulating influenza viruses already lack sensitivity to this drug group [45].

We have already shown an additive therapeutic effect of a combination therapy with intranasally applied iota-carrageenan and orally administered Oseltamivir in lethally H1N1 A/PR/8/34 infected mice and a treatment start 48 hours post infection (hpi) [37].

Due to these very promising results we further developed the concept of combining carrageenan with an NI therapy. In contrast to Oseltamivir, which needs to be activated by metabolic conversion, Zanamivir is directly applied as active drug and can also be administered intranasally [46–52]. The potential of an intranasal administration of Zanamivir was investigated by GlaxoSmithKline. In seven clinical challenge trials 66 volunteers were infected with influenza B/Yamagata/16/88 and 213 with influenza A/Texas/36/91 (H1N1). 156 of these participants got intranasally applied Zanamivir at different doses (daily dose levels from 6.4 mg to 96 mg) for prophylaxis or therapy [46,47,53,54]. These challenge trials showed that treatment starting before and up to 36 hours post virus inoculation was associated with prevention of laboratory confirmed influenza and febrile illness as well as a reduction in viral titers, duration of shedding and symptoms. In total, safety data from 1092 patients after intranasal application of Zanamivir were published and no evidence for Zanamivir induced adverse events or increased frequencies of local nasal intolerance in comparison to placebo groups was found [46,49,52].

Taken together, the combination of a carrageenan nasal spray that provides broad antiviral activity against upper respiratory infections—including influenza—with Zanamivir, a specific anti-influenza drug, meets the existing medical need to treat multiple viral infections. In the present work we investigate the therapeutic effect of a combination of carrageenan and Zanamivir *in-vitro* and in an animal model.

## Material and Methods

### Compounds

Kappa-carrageenan and iota-carrageenan were purchased from FMC Biopolymers (Philadelphia, PA). The identity, purity (>95%) of carrageenan subtypes and the molecular weight (>100,000) was confirmed by NMR analysis as described elsewhere [55] and the presence of lambda-carrageenan was below the detection limit of 3%. The dry polymer powders were dissolved in aqua bidest (Fresenius Kabi, Austria) to a final concentration of 2.4 mg/ml iota- and 0.8 mg/ml kappa-carrageenan. This 2x stock solution was sterile filtered through a 0.22 μm filter (PAA, Switzerland) and stored at room temperature until use. For further testing the stock solution was diluted to a mixture containing 1.2 mg/ml iota-carrageenan and 0.4 mg/ml kappa-carrageenan (hereinafter referred to as "carrageenan").

Zanamivir was purchased as powder (Haosun Pharma, China) and the identity and purity was confirmed by NMR analysis. Zanamivir was either dissolved in carrageenan or placebo solutions, followed by sterile filtration through a 0.22 μm filter (Sarstedt, Germany). For *in-vivo* studies all Zanamivir containing solutions were freshly prepared.

### Cells and Viruses

Madin-Darby canine kidney (MDCK) cells were obtained from the American Type Culture Collection (ATCC, Manassas, VA) and cultivated in a 37°C incubator (Sanyo, Japan; CO_2_: 5%, relative humidity: >95%). MDCK cells were grown in Dulbecco's minimal essential (DMEM) high glucose medium (PAA, Austria) supplemented with 10% fetal bovine serum (FBS; PAA, Austria; heat inactivated).

Influenza virus A/Hansa Hamburg/01/09 (H1N1(09)pdm) was kindly provided by Peter Staeheli Department of Virology, University of Freiburg, Germany and previously described in [56]; A/Teal/Germany/Wv632/05 (H5N1) previously published in [57] (accession numbers CY061882-9) and A/Turkey/Germany/R11/01 (H7N7) (taxonomy ID 278191, accession number AEZ68716) were supplied by courtesy of Martin Beer, Institute of Diagnostic Virology, Friedrich-Loeffler-Institute, Riems, Germany; A/Aichi/2/68 (H3N2) was purchased from the ATCC. All influenza viruses were propagated in MDCK cells at 37°C and 5% CO_2_ in influenza medium [Opti-Pro serum free medium (Gibco, Austria) supplemented with 4 mM L-glutamine (PAA, Austria), 1% antibiotic-antimycotic mix (PAA, Austria) and 5 μg/ml trypsin (Sigma Aldrich, Austria)].

### Evaluation of anti-influenza activity in a semi-liquid plaque assay

To determine the 50% inhibitory concentration (IC_50_) and the combination effect of carrageenan and Zanamivir, a semi-liquid plaque assay was developed. Into 96 well tissue culture plates 1.7x10^4^ MDCK cells/well were seeded and infected at 90% confluence (24–28 hours later). Serial dilutions of carrageenan and Zanamivir were prepared in assay medium (influenza medium without trypsin). For infection, viruses were diluted to an MOI of 0.003 (H1N1(09)pdm and H3N2 Aichi), 0.015 (H5N1) or 0.004 (H7N7), respectively, in assay medium and incubated at room temperature (RT) for 10 min with the serial dilutions of carrageenan and/or Zanamivir, respectively. For evaluation of the combination effect of carrageenan and Zanamivir, viruses were diluted in assay medium containing constant concentrations of either carrageenan or Zanamivir. The other substance was serially diluted and used for virus incubation. Cells were infected in 6 replicates/compound dilution, respectively, and incubated at RT for 45 min before inoculum removal. Cells were further incubated with the respective concentration of the investigated substances present in the overlay [influenza medium with 2.25% Carboxymethylcellulose (CMC, Fluka, Austria)] for 30–42 hours at 37°C. Evolving plaques were evaluated after methanol/acetone cell fixation by immune staining with antibodies either directed against the influenza A nucleoprotein (AbD Serotec, Germany) (for H1N1(09)pdm, H5N1 and H7N7) or the hemagglutinin (AbD Serotec, Germany) (for H3N2). Analysis was done with a HRP labeled detection antibody (Thermo Scientific, Germany) using TMB (Biolegend, Germany) as substrate and a microplate reader at 450 nm. The reduction of detected signal represents a reduction in the number and size of plaques and indicates suppression of viral replication during infection and cultivation.

After the immunostaining cells were stained with 0.005% crystal violet solution to assess the condition of the cell layer and the toxicity of the compounds. IC_50_ values and standard deviations were calculated for a sigmoidal dose response model using XLfit Excel add-in version 5.3.1.3.

### Mouse experiments

All animal experiments were carried out according to the guidelines of the “European Convention for the Protection of Vertebrate Animals used for Experimental and other Scientific Purposes” and the Austrian law for animal experiments. All animal experiments were approved by the Veterinary University of Vienna institutional ethics committee and performed under the Austrian Federal Ministry of Science and Research experimental animal license numbers BMWF-68.205/0262-II/3b/2011 and BMWF-68.205/0142-II/3b2012. C57BL/6 mice were purchased from Janvier Labs, France and maintained under standard laboratory conditions in the animal facilities of the Veterinary University of Vienna. For euthanasia and anesthesia asphyxiation through CO_2_ was used and all efforts were made to minimize suffering.

For infection experiments, 3–5 weeks old female mice were intranasally inoculated with 50 μl influenza virus solution (25 μl/nostril) containing 2.27x10^3^ or 1.65x10^3^ plaque-forming unit of H1N1(09)pdm or H7N7, respectively. Subsequently, treatment started 24, 48 or 72 hpi, as indicated for the different experiments. Treatment was performed intranasally either with 50 μl therapeutic solution or placebo twice per day for 5 days. As therapy either carrageenan (containing 1.2 mg/ml iota-carrageenan and 0.4 mg/ml kappa-carrageenan to provide a daily dose of 12 mg/kg body weight (BW)), Zanamivir (containing either 130 μg/ml or 390 μg/ml Zanamivir, to provide a daily dose of 1 or 3 mg/kg BW, respectively) or a combination of carrageenan and Zanamivir were used. Carrageenan and Zanamivir are used at non-toxic concentrations as shown by [58] and [59]. Mice were monitored twice daily for 15 days for survival and weight loss. Mortality also includes mice that were sacrificed for ethical considerations when they had lost more than 25% of their initial body weight. We confirm the viral infection in these animals by necropsy and scoring of the lung inflammation.

## Results

### Zanamivir and carrageenan exhibit different antiviral activity against individual influenza strains

As the mechanisms underlying the antiviral activity of NI and carrageenans are fundamentally distinct, they are likely to exhibit different activities towards the individual influenza virus strains. As a result, in combination they could complement each other to provide protection against a broader spectrum of influenza virus strains than the individual compounds.

To test this hypothesis, we investigated the sensitivity of various influenza virus strains to Zanamivir and carrageenan in an adapted plaque reduction assay with semi-liquid overlay in MDCK cells [60,61]. Using this method, we determined the IC_50_ of Zanamivir and carrageenan against influenza A viruses of human and animal origin, namely H1N1(09)pdm (A/Hansa Hamburg/01/09), H3N2 (A/Aichi/2/68), low pathogenic (LP) H5N1 (A/Teal/Germany/Wv632/05) and LP H7N7 (A/Turkey/Germany/R11/01) (Table 1). Both substances were non-toxic at the highest tested concentration (400 μM Zanamivir and 533 μg/ml carrageenan), neither was their combination. Furthermore, CMC in the overlay did not show any virus inhibitory effect (data not shown).

**Table 1:** IC_50_ values of carrageenan and Zanamivir for influenza A viruses of human and animal origin.

	IC_50_ Carrageenan^a^ [μg/ml]	IC_50_ Zanamivir^a^ [μM]
H1N1(09)pdm A/Hansa Hamburg/01/09	0.39 ± 0.03	0.19 ± 0.04
H3N2 A/Aichi/2/68	0.92 ± 0.05	15.93 ± 13.25
H5N1 A/Teal/Germany/Wv632/05	10.14 ± 1.66	0.18 ± 0.09
H7N7 A/Turkey/Germany/R11/01	118.48 ± 14.08	22.97 ± 5.76

^a^ IC_50_ values were calculated in comparison to untreated infected cells. Each value represents the mean IC_50_ of 6 replicates/assay and their standard deviation.

Inhibition of viral replication of all tested influenza strains was achieved with both substances. However, the IC_50_ values varied widely depending on the influenza virus strain. The IC_50_ values of Zanamivir ranged between 0.18 μM for H5N1 and 22.97 μM for H7N7 and that of carrageenan from 0.39 μg/ml to 118.48 μg/ml for H1N1(09)pdm and H7N7, respectively (see Table 1). These results demonstrate that carrageenan and Zanamivir target individual influenza strains to different extents so that they may complement each other to provide broader anti-influenza activity.

### Carrageenan and Zanamivir act synergistically against human and animal derived influenza A strains

**Fig 1:**
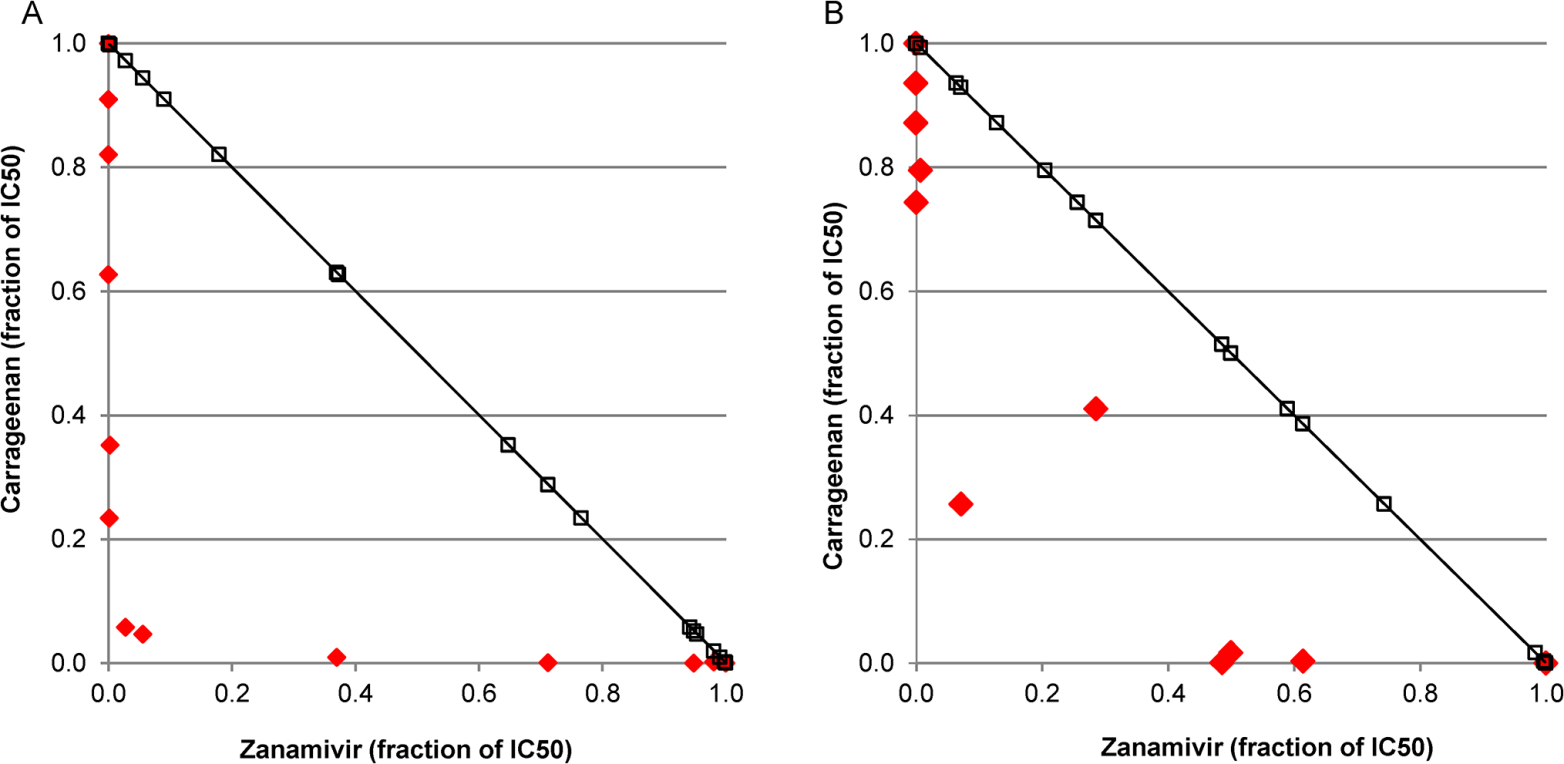
Isobologram of compound interaction. Comparison of the combination of different doses of both compounds necessary to reach 50% replication inhibition of (A) H7N7 and (B) H1N1(09)pdm (◆) to a model of dose additivity that would represent a curve progression of 1 (□). Dose response was tested with an adapted plaque reduction assay with semi-liquid overlay in MDCK cells. On the x-axis the concentration of Zanamivir and on the y-axis the concentration of carrageenan is presented. The concentrations (determined as mean of 3 replicates) are given as the fraction of the respective IC_50_ values of the different viruses with the particular compound (IC_50_ = 1).

The type of compound interaction was characterized by employing isobolograms (Fig 1). As described in [62], isobolograms graphically compare the doses of two compounds needed to reach 50% inhibition to the predicted doses calculated based on a model of drug additivity. A curve linearity of ~1 is expected for an additive compound interaction whereas a curve progression <1 argue for synergistic and >1 for an antagonistic compound interaction.

Two virus strains were selected for those experiments, one being the most sensitive to carrageenan (H1N1(09)pdm) and one being the least sensitive (H7N7). In both cases the isobolograms show a synergistic interaction of carrageenan and Zanamivir (Fig 1). Thus, it was shown that Zanamivir and carrageenan target individual influenza viruses with different efficiencies, most probably due to their different antiviral strategies. As a result, the combination provides synergistic activity with higher protection against a broader spectrum of influenza virus strains than the individual compounds.

### Intranasal treatment with the combination synergistically protects mice from lethal influenza H7N7 infection

In the influenza animal model, C57Bl/6 mice are challenged with a lethal dose of the respective virus and treated with different regimens in comparison to a vehicle control (placebo). Infection and treatment (twice a day for 5 days) are done intranasally without anesthesia. We investigated whether the combination of Zanamivir and carrageenan is more efficacious in reducing mortality than the corresponding mono-therapies.

**Fig 2:**
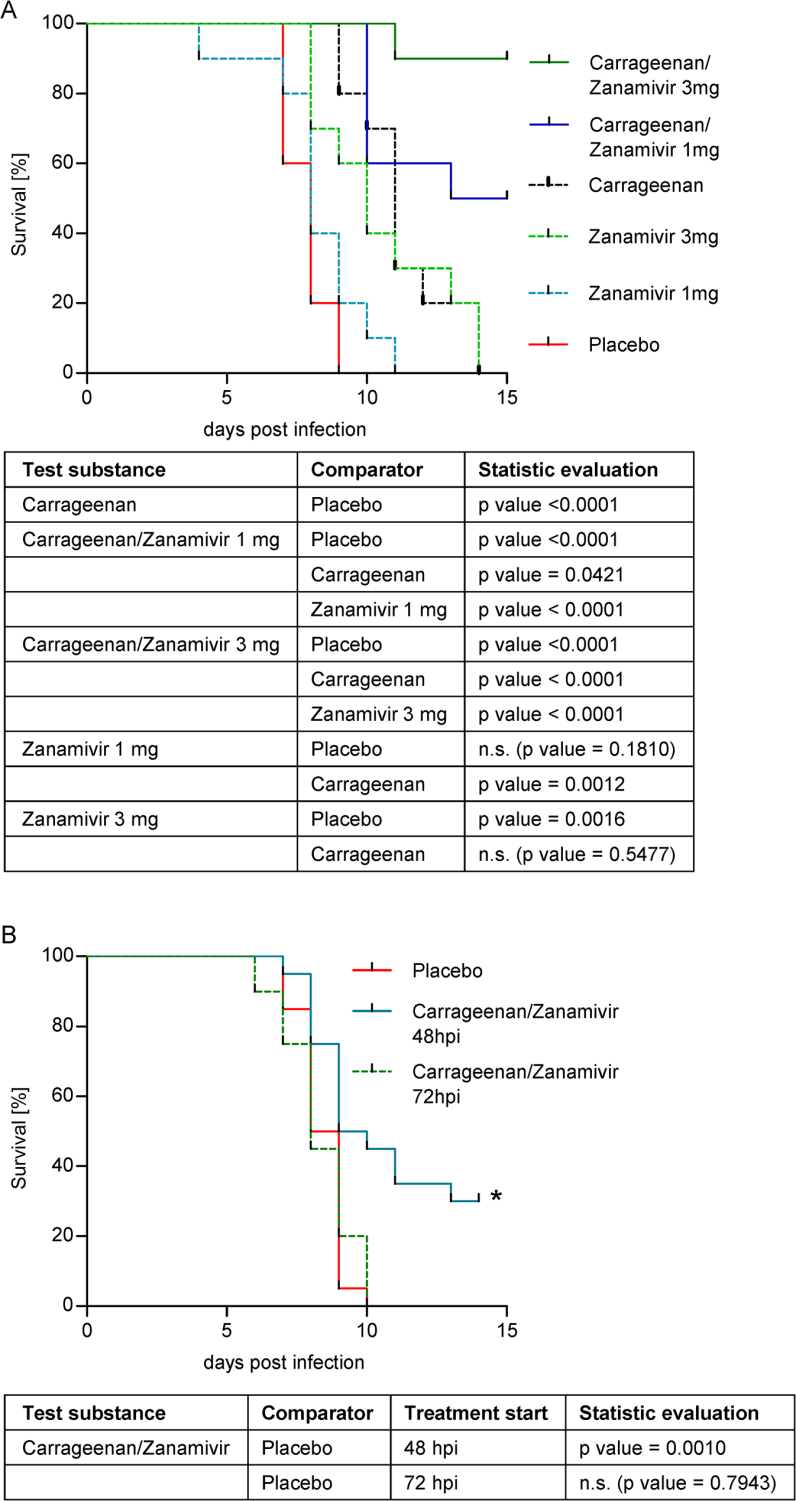
Therapeutic efficacy in influenza H7N7 lethally infected mice. (A) Mice (n = 10 per group) were lethally intranasally infected without anesthesia on day 0 and intranasally treated twice per day either with placebo or with the mono-therapies consisting of carrageenan (12 mg/kg BW/day) or Zanamivir (1 and 3 mg/kg BW/day) or a combination thereof. Treatment started 24 hpi and continued for 5 days. (B) Mice (n = 20 per group) were lethally intranasally infected without anesthesia on day 0 and intranasally treated twice per day either with placebo or a combination of carrageenan with Zanamivir (3 mg/kg BW/day). Treatment started either 48 hpi or 72 hpi and continued for 5 days. On the y-axis the survival of mice [%] and on the x-axis the time post infection [days] is given. Placebo treated uninfected control mice showed 100% survival in both experiments (data not shown). Statistical analyses were conducted using log rank test and are shown beneath the graphs. Values of p<0.05 were considered statistically significant; non-significance (n.s.) was obtained with p-values >0.05.

First, we determined the minimal effective dose of a Zanamivir mono-therapy that significantly improved survival time of H1N1 and H7N7 infected mice. For the H7N7 lethal infection the minimal effective dose of Zanamivir as mono-therapy ranged between 1 and 3 mg/kg BW/day (data not shown). Next, we compared the antiviral activity of carrageenan (12 mg/kg BW/day) and Zanamivir (1 and 3 mg/kg BW/day) mono-therapies with the respective combination versus placebo treatment. Survival rates of mice with treatment starting 24 hpi are shown in Fig 2A. All placebo treated mice died between day 7 and 9 and also in all mono-therapy groups 100% lethality was observed until day 15. In contrast, the combination therapies led to 50% and 90% survival, depending on the Zanamivir concentration. Statistical analysis showed that the Zanamivir mono-therapy 1 mg/kg BW/day did not show a significant benefit (p = 0.1810), whereas the mono-therapy with 3 mg/kg BW/day significantly increased the survival rate compared with placebo treated mice (p = 0.0016). Both Zanamivir concentrations experienced significant benefit in survival by the combination with carrageenan (p<0.0001). Similarly, the combination therapies resulted in remarkably increased survival (p = 0.0421 for 1 mg and p<0.0001 for 3 mg/kg BW/day) when compared to the carrageenan mono-therapy. No statistically significant difference was observed between the combination containing 3 mg/kg BW/day Zanamivir and that containing 1 mg/kg BW/day (p = 0.0525). However, a trend for an increased survival rate with the higher Zanamivir concentration was evident. Therefore, for further investigation the combination therapy containing 3 mg/kg BW/day Zanamivir was evaluated in lethally H7N7 infected mice.

Next, the therapeutic potential of the combination with a delayed therapy start 48 or 72 hpi versus placebo treatment was explored. The survival rates of mice are shown in Fig 2B. All placebo treated mice died until day 10 and also in the group with the treatment start 72 hpi 100% lethality was found. In contrast, the combination therapy starting 48 hpi provided a statistically significant enhanced survival rate in comparison to placebo-treated mice (p = 0.0010).

In summary, the combination of two effective, established mono-therapies resulted in a significantly enhanced survival in lethally H7N7 infected mice. Additionally, the combination therapy was highly efficient in comparison to placebo treatment even after a treatment onset up to 48 hpi.

### Intranasal therapy with carrageenan and Zanamivir starting 72 hpi significantly protects lethally influenza H1N1(09)pdm infected mice

Next, the minimal effective dose of Zanamivir used as mono-therapy was evaluated in a lethal H1N1(09)pdm mouse model, following the same scheme as described in the H7N7 experiments. The lowest effective dose of Zanamivir after a treatment start 24 hpi was 1 mg/kg BW/day and its combination with carrageenan was highly effective (data not shown). In the following experiment the therapeutic potential of the combination with a therapy start 48 or 72 hpi was investigated in comparison with the respective placebo treatment.

**Fig 3:**
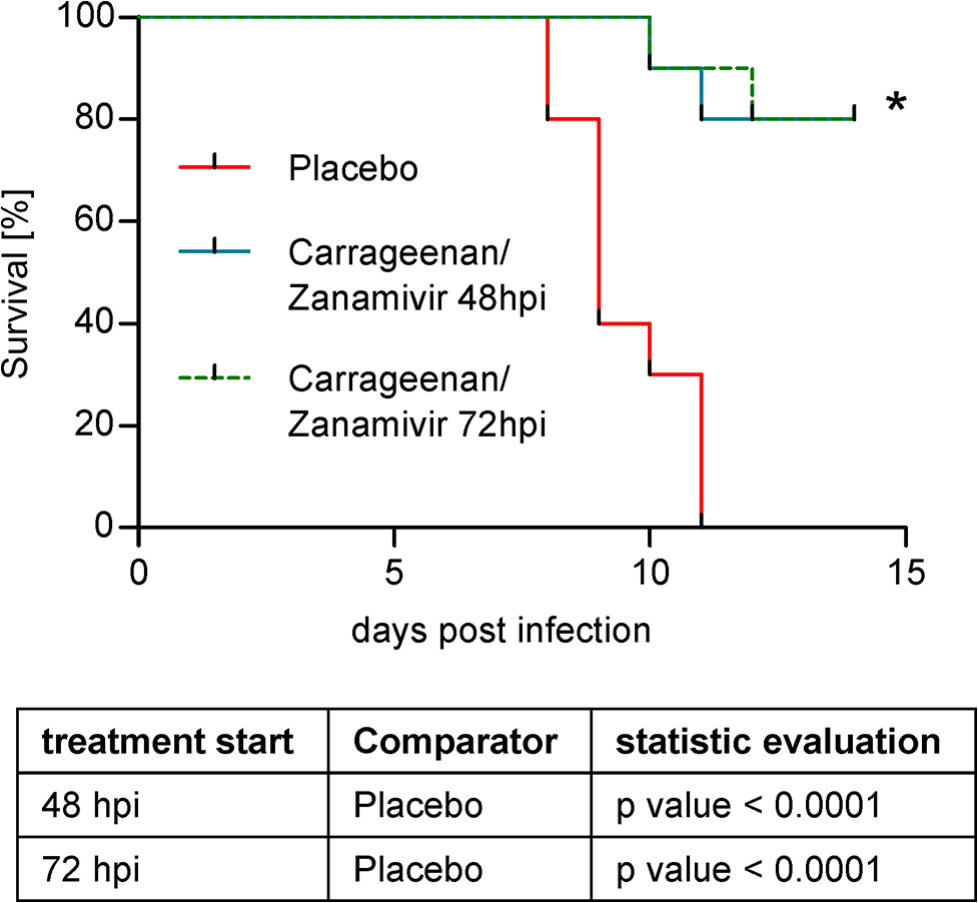
Therapeutic efficacy in influenza H1N1(09)pdm lethally infected mice. Mice (n = 20 per group) were lethally intranasally infected without anesthesia on day 0 and intranasally treated twice per day either with placebo or a combination of carrageenan and Zanamivir (1 mg/kg BW/day). Treatment started either 48 hpi or 72 hpi and continued for 5 days. On the y-axis the survival of mice [%] and on the x-axis the time post infection [days] is given. Placebo treated uninfected control mice showed 100% survival (data not shown). Statistical analyses were conducted using log rank test and are shown beneath the graphs. Values of p<0.05 were considered statistically significant.

As shown in Fig 3, the survival rates of mice treated with the combination therapy were highly significantly increased in comparison to the placebo group (p<0.0001). There was no difference in survival between the two therapy starting points, 48 or 72 hpi, which both resulted in 80% survival on day 15. Subsequent experiments to investigate the effect of a treatment start 96 hpi showed no significantly enhanced survival over placebo treatment (data not shown).

## Discussion

We investigated the antiviral effect of a combination of carrageenan with the NI Zanamivir in cell culture studies and in mouse influenza infection models. We have previously shown that a combined therapy of iota-carrageenan with the NI Oseltamivir led to significantly enhanced survival in mice infected with H1N1 PR/8/34 in comparison with the respective mono-therapies [37]. However, Oseltamivir is an orally administered prodrug, which has to be converted into its active form by metabolic processing. Therefore, a further development of a combination nasal spray was not possible with Oseltamivir. Instead Zanamivir–a NI that is applied as active drug—was chosen for the development of a compound combination.

During the evaluation process we found that the binding efficiency of different carrageenan subtypes on different influenza strains varies. The combined use of iota- and kappa-carrageenan for the treatment of lethally influenza infected C57Bl/6 mice revealed a better therapeutic effect than the use of iota-carrageenan alone (S1 Fig). Thus, to provide a broader spectrum of activity against different influenza virus strains, a mixture of iota- and kappa-carrageenan (designated as carrageenan) was used for further evaluation.

For investigation of the effect of a compound combination of carrageenan and Zanamivir, we examined their inhibition efficiency, individually and in combination, against influenza viruses in an adapted plaque reduction assay with semi-liquid overlay in MDCK cells. The combination showed a synergistic inhibition of virus replication in *in-vitro* assays with all tested influenza viruses (Fig 1). This indicates that the physical interaction of the polymer with the virus does not disturb the inhibition of the neuraminidase by Zanamivir. This was confirmed in *in-vitro* tests examining a potential influence of the polymer on the neuraminidase inhibiting activity of Zanamivir (data not shown). Hence, the observed synergistic effect is based on the combination of two distinct underlying mechanisms. As a result, in the proposed combination both mechanisms would complement each other to provide more efficient protection against a broader spectrum of influenza virus strains than the individual compounds.

The synergistic effect was also shown in lethal mice models (Fig 2 and Fig 3). The pathogenicity of influenza viruses in mice varies and is dependent on the strain and its adaptation to the host. Depending on virus dose and strain, influenza viruses can induce lethal infections in certain mouse strains usually within two weeks [37,63]. In our model, C57Bl/6 mice are challenged intranasally with a lethal dose of the respective virus and treated with different regimens in comparison to a vehicle control (placebo). In such a model, early virus replication takes place in the upper respiratory tract. From there, virus spreads to the lung and causes lethal pneumonia. The effect of the treatment on mortality is assessed in comparison to placebo-treated control mice. Of all *in-vitro* tested influenza strains the H1N1(09)pdm and the LP H7N7 are particularly interesting for two reasons. First, they are highly relevant pathogens, as both are involved in recent influenza outbreaks. The H1N1(09)pdm is associated with more than 18,400 deaths in the season 2009/2010 while the LP H7N7 carries an HA closely related to that of the avian influenza H7N9 virus which has caused more than 175 deaths until October 2014 [64]. Second, they are of special interest for the carrageenan/Zanamivir combination approach. They have shown to differ in *in-vitro* susceptibility to carrageenan, Zanamivir (Table 1) and the combination thereof (Fig 1). While H1N1(09)pdm was highly sensitive to inhibition by both substances alone, H7N7 required much higher concentrations of carrageenan and Zanamivir, respectively, to achieve similar inhibition efficiencies. Therefore, both virus strains were chosen to further explore the efficiency of the combination therapy in a mouse model.

We established lethal mouse models with both viruses that resulted in 6.8 and 8.5 mean survival days for LP H7N7 and H1N1(09)pdm, respectively. These results are in good accordance to similar already published lethal influenza models [65–67]. In our models the lowest effective dose for Zanamivir at a treatment start 24 hpi was found to be between 1 to 3 mg/kg BW/day for both viruses. This concentration range is relatively high in comparison to other published studies. However, these studies were done under anesthesia with different viruses and a prophylactic therapy start [65,66]. The fact that a higher dose of NI is needed for an effective treatment when the therapy starts 24 hpi is already known for Oseltamivir [68]. Nonetheless, also data with much higher effective concentrations (≥10 mg/kg BW/day [69]) and with similar concentrations of Zanamivir (2.5 mg/kg BW/day [67]) were published as well.

We found that the combination of carrageenan with 3 mg/kg BW/day Zanamivir used for treatment of H7N7 infected mice resulted in significantly enhanced survival of mice in comparison to both mono-therapies (Fig 2). The significantly enhanced survival compared to the placebo treated group was also found after a delayed treatment start 48 hpi. Furthermore, in the H1N1(09)pdm model the combination of carrageenan with 1 mg/kg BW/day Zanamivir showed statistically significant enhanced survival in comparison to placebo treatment even after a treatment start 72 hpi. This is a remarkable finding since NIs are normally not effective when applied 72 hpi.

The finding supports the development of the Zanamivir and carrageenan combination approach. As the intranasal treatment regime is incapable to effectively treat virus infections of the lung, the primary target of such a product is the prophylaxis and therapy of uncomplicated influenza. Since the majority of influenza infections causes uncomplicated illnesses and practically all cases of influenza start with an infection of the nasal cavity or the upper respiratory tract, the therapeutic potential is huge. However, clinical studies are required to elucidate and demonstrate the potential of the proposed combination therapy.

Combination of antiviral strategies has led to impressive achievements in the combat against other viral disease like HIV. In particular the problem of antiviral resistance could be addressed with this strategy. In the last decade concerns have been raised about the increased emergence of Oseltamivir resistant influenza viruses. The augmented appearance of viruses carrying the mutation H275Y in the neuraminidase of H1N1(09)pdm viruses that confers resistance to Oseltamivir left Zanamivir as only treatment option for symptomatic patients infected with an Oseltamivir resistant influenza strain [70]. In contrast to Oseltamivir, resistance to Zanamivir is less frequent. To date, Zanamivir resistant influenza has been detected only once, in an immunocompromised patient [71,72]. However, lessons should be learned from previous anti-influenza interventions which resulted in occurrence of resistance against currently approved drugs [73]. Therefore, concerns are comprehensible that an increased Zanamivir use may also lead to the rapid emergence of resistances [74]. To overcome this threat, a combination of antivirals which inhibits virus replication by distinct mechanisms is a valid strategy. We checked for the possibility of generating double compound escape mutant viruses while passaging viruses in the presence of increasing concentrations of compound combinations. After 10 passages in MDCK cells no resistance to the compound combination for any tested influenza virus could be found (data not shown). However, this finding does not guarantee that emergence of Zanamivir escape mutants can be completely halted.

In summary, we demonstrated that the anti-influenza mechanisms of both single compounds complement each other. The combination provides synergistically better protection against a broader spectrum of influenza viruses than the individual compounds.

## Conclusions

A nasal spray containing carrageenan together with Zanamivir provides an easy to apply treatment of upper respiratory tract infections in patients under suspicion to be influenza infected. Patients would benefit from the fast and efficient treatment of uncomplicated influenza in the upper respiratory tract. Due to the faster influenza virus clearance from the upper respiratory tract and the independent antiviral mechanism of carrageenan and Zanamivir the likelihood to develop escape mutations against Zanamivir will be reduced. Both individual compounds are able to reduce severity and/or duration of the influenza illness and a combination is expected to work similarly. Additionally, due to the broad antiviral effectiveness of carrageenan, patients will receive in parallel a treatment of concomitant viral infections. Therefore, patients will benefit from a decreased probability to develop complications. In consideration of the complications known to accompany an influenza virus illness this combinational therapy meets an urgent medical need.

A second scope of this combination is the protection against newly emerging pandemic viruses during the time until identification of the virus followed by manufacturing and distribution of vaccines [43]. Even if, due to new reverse genetic techniques, less time for production of vaccines is needed, it still takes months before large quantities of vaccine are available [75]. During this time the human population should be protected to decelerate viral spread. At the moment the only available opportunities for personal protection are hygiene measures and the use of Tamiflu (brand name of Oseltamivir).

Novel protection and treatment options for influenza are desperately needed. Based on our encouraging results in mice we suggest testing a nasal spray containing carrageenan in combination with the neuraminidase inhibitor Zanamivir in clinical trials for prevention or treatment of uncomplicated influenza infections.

## Supporting Information

S1 Fig Therapeutic efficacy of iota-carrageenan solely or together with kappa-carrageenan in influenza H7N7 lethal infected mice. Mice (n = 20 per group) were lethally intranasally infected without anesthesia on day 0 and accordingly intranasally treated twice per day either with placebo or with iota-carrageenan or with a mixture of iota- and kappa-carrageenan. Treatment started 24 hpi and continued for 5 days. On the y-axis the survival of mice [%] and on the x-axis the time post infection [days] is given. Placebo treated, uninfected control mice showed 100% survival (data not shown). Statistical analyses were conducted using log rank test and are shown beneath the graphs. Values of p<0.05 were considered statistically significant; non-significance (n.s.) was obtained with p-values >0.05.
(TIFF)
