# Supplementary figures and images for: The Intranasal Application of Zanamivir and Carrageenan Is Synergistically Active against Influenza A Virus in the Murine Model
Source: PLoS One. 2015 Jun 8;10(6):e0128794. doi: 10.1371/journal.pone.0128794 (PMC4459876; doi:10.1371/journal.pone.0128794)

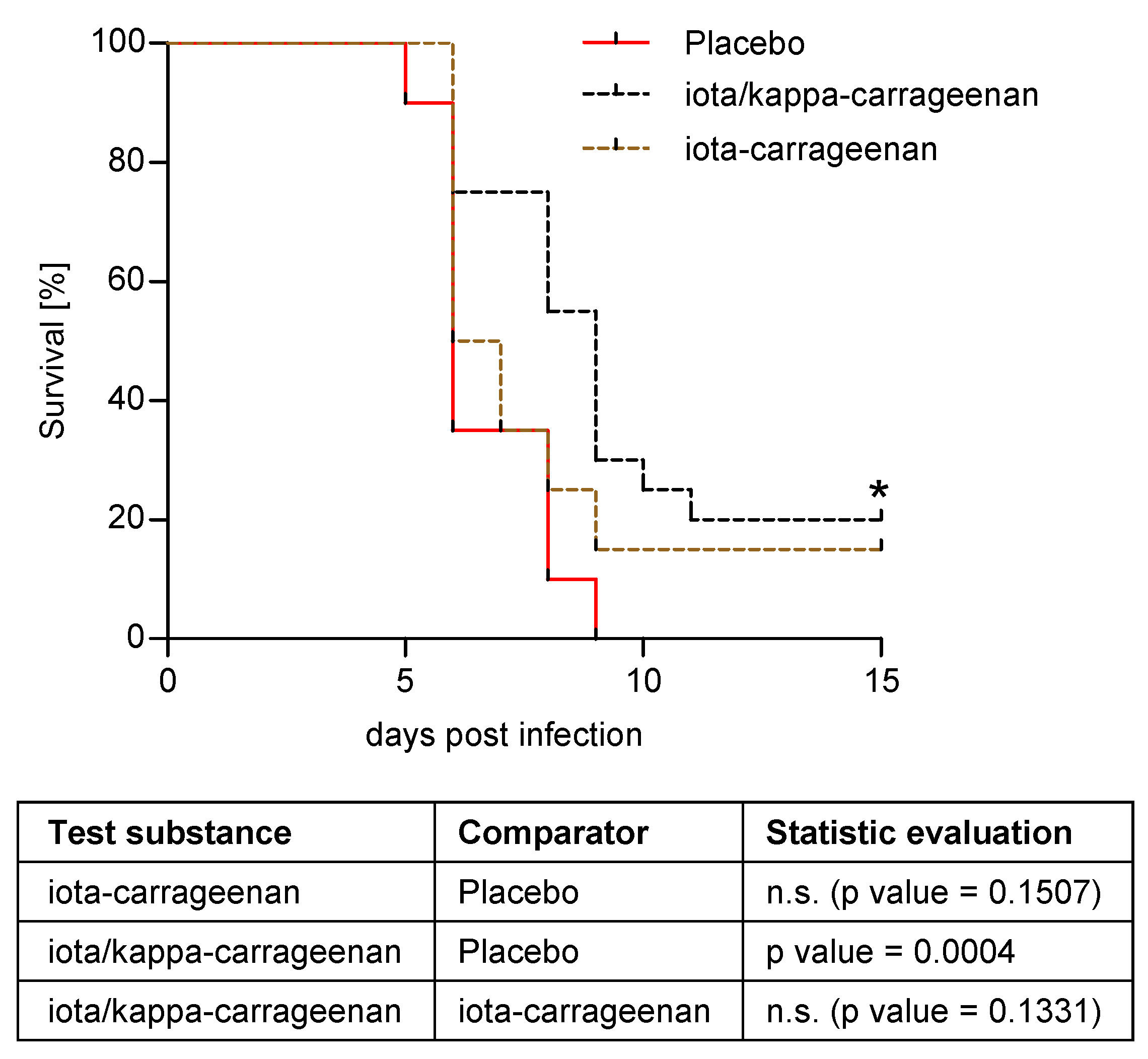

Supplement: S1 Fig — Mice (n = 20 per group) were lethally intranasally infected without anesthesia on day 0 and accordingly intranasally treated twice per day either with placebo or with iota-carrageenan or with a mixture of iota- and kappa-carrageenan. Treatment started 24 hpi and continued for 5 days. On the y-axis the survival of mice [%] and on the x-axis the time post infection [days] is given. Placebo treated, uninfected control mice showed 100% survival (data not shown). Statistical analyses were conducted using log rank test and are shown beneath the graphs. Values of p<0.05 were considered statistically significant; non-significance (n.s.) was obtained with p-values >0.05. (TIFF) [file pone.0128794.s001.tiff]
